# Non-Coding Variants in *BRCA1* and *BRCA2* Genes: Potential Impact on Breast and Ovarian Cancer Predisposition

**DOI:** 10.3390/cancers10110453

**Published:** 2018-11-16

**Authors:** Elizabeth Santana dos Santos, François Lallemand, Leslie Burke, Dominique Stoppa-Lyonnet, Melissa Brown, Sandrine M. Caputo, Etienne Rouleau

**Affiliations:** 1 A.C.Camargo Cancer Center, São Paulo 01509-010, Brazil; elizabeth.santanadossantos@gmail.com; 2 Department of Oncology, Center for Translational Oncology, Cancer Institute of the State of São Paulo—ICESP, São Paulo 01246-000, Brazil; 3 Department of Genetics, Institut Curie, 75005 Paris, France; francois.lallemand@curie.fr (F.L.); dominique.stoppa-lyonnet@curie.fr (D.S.-L.); sandrine.caputo@curie.fr (S.M.C.); 4 Institut Curie, Paris Sciences Lettres Research University, 75230 Paris, France; 5 School of Chemistry and Molecular Biosciences, The University of Queensland, Brisbane, QLD 4072, Australia; l.burke@uq.edu.au (L.B.); melissa.brown@uq.edu.au (M.B.); 6 Department of Genetics at Institut Curie, Université Paris Descartes, 75006 Paris, France; 7 INSERM U830, Institut Curie, 75248 Paris, France; 8 Institut Gustave Roussy, 94805 Villejuif, France

**Keywords:** *BRCA1*, *BRCA2*, non-coding variants, promoter, hereditary breast cancer, hereditary ovarian cancer

## Abstract

*BRCA1* and *BRCA2* are major breast cancer susceptibility genes whose pathogenic variants are associated with a significant increase in the risk of breast and ovarian cancers. Current genetic screening is generally limited to *BRCA1*/*2* exons and intron/exon boundaries. Most identified pathogenic variants cause the partial or complete loss of function of the protein. However, it is becoming increasingly clear that variants in these regions only account for a small proportion of cancer risk. The role of variants in non-coding regions beyond splice donor and acceptor sites, including those that have no qualitative effect on the protein, has not been thoroughly investigated. The key transcriptional regulatory elements of *BRCA1* and *BRCA2* are housed in gene promoters, untranslated regions, introns, and long-range elements. Within these sequences, germline and somatic variants have been described, but the clinical significance of the majority is currently unknown and it remains a significant clinical challenge. This review summarizes the available data on the impact of variants on non-coding regions of *BRCA1/2* genes and their role on breast and ovarian cancer predisposition.

## 1. Hereditary Breast and Ovarian Cancer (HBOC) Syndrome

Breast cancer is the most common cancer among women worldwide and ovarian cancer is the deadliest gynecological cancer. Mutations in high risk genes contribute to at least 10% and 15% of breast and ovarian cancer diagnoses, respectively, with cases frequently associated with a strong family history and early onset of disease. Hereditary Breast and Ovarian Cancer (HBOC) Syndrome is an autosomal dominant syndrome, which is caused primarily by germline mutations in two genes: breast cancer susceptibility gene 1 (*BRCA1*), which was first described in 1994, and breast cancer susceptibility gene 2 (*BRCA2*), which was discovered one year later [1,2]. This syndrome is characterized by an increased risk for female and male breast cancer, ovarian cancer, and to a lesser extent, other cancers, such as prostate cancer, pancreatic cancer, and melanoma. For a heterozygous carrier, it has been reported that the lifetime risk is as high as 70% for breast and 20–40% for ovarian cancer [3,4,5]. Therefore, prophylactic surgeries, such as bilateral mastectomy and salpingo-oophorectomy, are effective risk reduction strategies.

Beyond the preventive aspects, understanding the mechanism of predisposition can help in the choice of treatment to improve the response and survival of patients. Advances in translational research have confirmed the biological and preclinical evidences making it increasingly apparent that *BRCA1/2* mutations are biomarkers that may predict the clinical response of breast and ovarian cancer patients to platinum salts and poly (ADP-ribose) polymerase (PARP) inhibitors [6,7,8,9,10,11]. Therefore, mutational status is becoming increasingly important for the management of BRCA related cancers as PARP inhibitors and *BRCA1/2* mutation targeting seem to be a hopeful approach for this group of patients. After three decades of research, several other breast cancer susceptibility genes have been identified, but with lower penetrance and associated risk [12,13].

The *BRCA1* gene encodes a nuclear protein of 1863 amino acids [2]. This protein contains a RING domain in the N-terminal region and two BRCT domains in its C-terminal region, through which it interacts with multiple partners, performing a variety of cellular functions that are particularly related to the DNA damage repair [14,15,16,17]. The *BRCA2* gene also encodes a nuclear protein that is composed of 3418 residues [1]. *BRCA2*, like *BRCA1*, is involved in DNA repair by homologous recombination, and it interacts with different partners (such as RAD51 and PALB2) to maintain the stability of the genome [18]. For the moment, three BRCA2 regions have been described as particularly important for homologous recombination function: N-terminal PALB2-binding site, BRC repeats (which correspond to eight consecutive motifs located in the central region of the protein and constitutes the principal RAD51 interaction site), and the C-terminal region (composed of three oligosaccharide binding folds, a helical domain, and a tower domain that together constitute the DNA binding region and contain a RAD51 binding domain) [18,19].

Although sequencing of these high penetrance genes *BRCA1/2* has been available for over 20 years, after two decades of intense research, a pathogenic variant is identified in approximately 10% of tested families [20]. Despite the remarkable advances seen in the past years, for the majority of HBOC families, little is understood about the underlying molecular mechanisms of cancer susceptibility. New technologies are being developed to extensively search in parallel for a pathogenic variant in a panel of other genes related to the syndrome. These high to moderate penetrance variants in known breast cancer related genes, such as *TP53*, *PTEN*, *STK11*, *CDH1*, *ATM*, *BRIP1*, *PALB2*, and *RAD51* isoforms (*RAD51C*, *D*, *B*) may also contribute to hereditary predisposition, but altogether these variants only explain about 5% of the unsolved cases [21]. Additional attempts to identify breast cancer risk genes have uncovered a large number of low risk loci that generally map to gene regulatory regions. The remainder of the risk is therefore likely to be a combination of not yet identified high, moderate, or low risk variants located in the non-coding regions of the aforementioned genes or in currently unidentified breast cancer risk loci. It is noteworthy that current *BRCA1/2* routine screening is limited to the coding region and intron/exon boundaries. However, protein inactivating mutations may not be the only mechanism by which their function is altered. Reduction in gene expression by changes in trans acting factors (TFs) or cis-regulatory regions may achieve the same end as truncating mutations in the gene itself. Since limited information currently exists about the impact of variants in *BRCA1/2* non-coding regions, the majority of variants that were identified in these regions remain unclassified. Therefore, about 80% of *BRCA1/2* gene screening remains negative, while introns and proximal untranslated regions remain relatively unexplored. However, evidence of non-coding variants impact on cancer risk and response to treatment begin to emerge [22].

Current technological sequencing advancements and development of bioinformatics tools has enabled the exploration and elucidation of the genome structure and non-coding DNA regions. The description of the functional elements of the human genome by the encyclopedia of DNA elements provided a better understanding of the human genome expression regulation and how regulatory data is encoded. This effort demonstrated that most of the human genome is involved in gene expression regulation, while the small minority of the nucleotides (1.2%) encodes proteins within humans. The ENCODE project has also described thousands of regulatory active regions and showed that 90% of common variants fall outside the coding regions of the genes [23]. Nevertheless, the majority of the studies to date have focused on the coding regions of the cancer related genes.

This article summarizes current knowledge of non-coding regulatory *BRCA1/2* regions and the variants that are located in these regions.

## 2. Germline Cancer-Associated Variants in the Regulatory Regions

Until recently, most attention had been focused on the coding regions of the genes that are associated with cancer risk. Exome sequencing of human genome and co-segregation studies have made evident that lots of disease-associated variants play a role in hereditary susceptibility. Since coding changes do not explain all of the predisposition cases, the importance of the non-coding regions (including promoters, introns, intergenic sequences, and non-coding RNAs) in biological functions and hereditary predisposition must be considered.

Gathered evidence indicates that genetic variants in the non-coding but functional elements can contribute to the development of hereditary cancers. The presence of variants in these regions can impact gene transcription by the creation or disruption of transcription factors binding sites, or by interfering with CpG island methylation which leads to an aberrant methylation pattern. In addition, variants may have an impact at the post-transcriptional level, creating or disrupting microRNA 3′ complementary binding sites in 3′UTRs, and interfering with the stability of RNAs and microRNAs. Moreover, the elucidation of three-dimensional (3D) chromatin structure reveals a complex network of interactions within the regulatory regions of the genome that includes long-range interactions between functionally coordinated domains lying hundreds of kilobases upstream or downstream of their target [24,25]. Therefore, non-coding sequence alterations may also influence this model of regulation. 

As non-coding sequences correspond to 98% of the genome, the identification of regions with a greater chance of bearing a variant that contributes to disease should be prioritized. Since transcriptional activity is correlated with less condensed chromatin regions, regulatory elements are often located in DNAse I hypersensitive sites. Furthermore, regions that are conserved in mammals, containing multiple binding sites for known transcription factors are most likely to be functional and present a higher probability of containing disease-associated variants [23]. Bioinformatics, experimental, and population-based approaches are complementary in identifying and validating key regulatory regions of the genome.

There is increasing data associating germline non-coding variants with cancer risk. Additionally, most cancer-associated single nucleotide variants (SNVs) that were identified through genome-wide association studies are located in non-coding regions, some of them with a proven role in gene expression regulation [26,27]. As examples: (i) a germline variant in the promoter of *TERT* (telomerase reverse transcriptase) gene (c.-57T>G) significantly increased promoter activity. This variant co-segregated with cancer in a family with 14 melanoma cases who were not carriers of germline mutations in the two known melanoma genes, *CDKN2A* and *CDK4* [28]. The variant increases *TERT* expression, probably by the creation of a new binding site for Ets, Elk1, and Elk4 transcription factors. The increase of *TERT* expression is a fundamental requirement for cell transformation and immortality [29,30]. (ii) Constitutional germline mutations have also been described in *MLH1* and *PTEN* promoters and correlated with the risk of cancer [31,32,33,34]. Interestingly, the 5′UTR *MLH1* variant c.-27C>A is an example of a non-coding sequence change associated with an epigenetic modification. The presence of the variant generates aberrant methylation of the promoter and silencing of the affected allele [31,32,34]. (iii) Additionally, it was proved that an enhancer region, which is located in the intergenic sequence on chromosome 8q24, interacts with MYC proto-oncogene, even though it is located 335kb from this gene. A variant located there (rs6983267) is associated with colorectal cancer risk via the disruption of transcription factor 7-like2 (TCFL2) binding site, a co activator of the Wnt-β catenin pathway [35].

A priority now is to identify the full spectrum of non-coding variants that contribute to disease and then determine their impact of gene function and disease risk. Indeed, as subtle quantitative effects are expected, it is challenging but important to define a threshold of effect that classifies these non-coding variants as “pathogenic variants” to allow for accurate genetic counseling.

## 3. Regulatory Regions in *BRCA1* and *BRCA2* Genes

*BRCA1* and *BRCA2* expression are controlled at the transcriptional and post-transcriptional levels. The key transcriptional regulatory elements are housed in gene promoters, introns, and long-range elements, while the key post-transcriptional control elements are predominantly located in 5′ and 3′ untranslated regions (UTRs). Both genes are expressed in a cell cycle regulated manner, with low levels of proteins being observed in G0 and early G1 phases before entry into S phase, and high levels are maintained through the S and G2 phases of the cell cycle [36,37].

*BRCA1* is a tumor suppressor gene that is located on chromosome 17q21 involved in DNA error-free repair by homologous recombination. The core promoter of *BRCA1* includes the non-coding exon 1 and part of intron 1 of *BRCA1*, as well as the exon 1 and part of intron 1 of the neighboring gene *NBR2* (chr17: 43,168,800–43,172,601). BRCA1 expression is complex with its transcription controlled by two different promoters, α and β, located upstream from the alternative first exon 1A (121bp) and 1B (378bp), respectively. These two promoters encode 5′UTR-a and 5′UTR-b [38,39], which share the same translation start codon (located in exon 2). These transcripts differ by the 5′UTR (exon 1) and they are expressed in a tissue specific fashion: exon 1B is only expressed in breast cancer while exon 1A transcripts are present in both normal or tumor tissue. The maintenance of the correct ratio between the two transcripts has the potential to be important for normal regulation and function. In vitro studies show that this structural difference is related to a lower translation efficiency of 5′UTR-a in comparison with 5′UTR-b [40].

The more efficient *BRCA1* promoter (α) consists of a region of 200 base pairs, upstream of the start site, which functions as a bidirectional transcriptional element able to direct expression in either the *BRCA1* or *NBR2* direction. There is some evidence to suggest that these two genes, separated by little more than 200 bp, are reciprocally regulated and present divergent transcription [41]. However, gene expression data from TCGA confirm the co-expression regulation for ovarian serous carcinomas but not in the breast cancer data set [42,43]. *BRCA1* promoter contains: a RIBS element that acts as an activator and possesses multi subunit EtsGA-binding protein binding sites [44], a CREB binding site that is a strong positive transcriptional element [45], a CAAT box [39], and an E2F binding site [46]. Since no estrogen responsive element (ERE) was identified in *BRCA1* promoter α, the stimulation of *BRCA1* expression by estrogen seems to result from an indirect effect of estrogen. In contrast, an ERE was described in *BRCA1* promoter β, so, in this case, the estrogen stimulation effect is due to estrogen bound to the DNA and a subsequently interaction with the transcription machinery to stimulate transcription [39,47]. In addition to promoter elements, upstream repressor elements were also described in regions upstream of the start of transcription and translation [48]. 

Gene promoter methylation has been proposed as an alternative mechanism for the transcriptional silencing of cancer-associated genes [49]. As a typical example, epigenetic silencing of *MLH1* that is associated with inherited variants leading to promoter methylation was described in familial colorectal cancers [32,50,51,52]. *BRCA1* promoter methylation appears to be more relevant for sporadic than for hereditary breast and ovarian cancers [53,54,55]. It is an uncommon event among BRCA mutation carriers. For the *BRCA1* gene, it was detected in about 3%and 11% of breast [56] and ovarian carcinomas [43], respectively.

There is limited information about regulatory elements outside of the *BRCA1* promoter. Suen and Goss localized a 36-bp repressor element in the first intron of *BRCA1* [48]. Wardrop and Brown subsequently described two evolutionarily conserved regions rich of TF binding sites in the second *BRCA1* intron that mediates both the activation and repression of the *BRCA1* gene [57]. In addition, we reported recently the enhancer property of an intronic sequence that is located in the intron 12 of *BRCA1* [58]. The *BRCA1* 3′ untranslated region (3′UTR) has been shown to be important for post-transcriptional regulation and this has been exemplified by a variety of variants located there that negatively regulate mRNA translation, probably by the disruption or creation of complementary MicroRNAs binding sites [59,60,61,62].

*BRCA2* is also a tumor suppressor gene that is located on chromosome 13ql2.3 [1]. Its core promoter was first described four years after *BRCA2* gene cloning [63]. It is located −66 to +129 from the transcriptional start site, and corresponds to a region rich in CG nucleotides and with several TF binding sites, including E-box, Ets/E2F, and SP1. *BRCA2* promoter is induced by NFκB and Elf1 [63,64], while repressed by P53, PARP1, and SLUG [65,66,67]. Recently, functional studies that were based on micro deletions mapped other regulatory promoter regions with up and down-regulating elements [68]. Like *BRCA1*, *BRCA2* is expressed in a cell cycle-regulated manner and the estrogen induction is also an indirect effect of mitogenic activity. Low protein levels are observed in G0 and early G1 phases, while peak levels are reached in late G1, S, and G2 phases of the cell cycle. Misra et al. described the bi-directional activity of *BRCA2* promoter, similar to that of *BRCA1*. It was shown that the forward and reverse promoter activity regulates both *BRCA2* and *ZAR2* transcription, respectively. Interestingly, during the G0 and G1 phase of cell cycle, this promoter is 8–20 times more active in the reverse orientation, increasing the production of the ZAR2 protein that binds to the promoter and silencing *BRCA2* expression. Whereas, during the pre-division phases (S/G2), the forward activity is 5–8 times higher and the ZAR2 is trapped in the cytoplasm [37]. Nevertheless, TCGA gene expression data does not confirm this co-expression regulation in the breast cancer data set, while no data is available for ovarian serous carcinomas [42,43].

Evidence suggests that promoter hypermethylation is not an obvious contributor to *BRCA2* related cancers [56]. For now, little information about *BRCA2* non-coding regions is available. A few cis-acting intronic polymorphisms that alter the binding of transcription factors at regulatory sites have been described [69], as well as one 3′UTR variant (*BRCA2*c.*172G>A), but with no clear evidence of pathogenicity [60].

## 4. Methods to Assess the Pathogenicity of *BRCA1/2* Non-Coding Variants

Variants classification is based on multiple lines of evidence, with population, computational, pathological, functional, and segregation data all being taken into account. Multifactorial prediction models are able to incorporate these different sources of data to calculate the probability of the variant being pathogenic. Currently, virtually all *BRCA1/2* non-coding variants are considered to be variants of uncertain significance (VUS) [70,71], as the prediction models have been built to classify variants that have a significant impact on protein function and they are generally associated with a high risk of disease. Multiple approaches are being applied to evolve in the classification of these variants. Individually, these resources are not enough for variant classification, but taken together, they may allow a better clinical interpretation.

### 4.1. In Silico Tools and Genetic Data

A variety of prediction software is available to evaluate the impact of variants on splicing and on the structure of the protein. However, since many tools are based on the effect of the protein, it is difficult to extrapolate this analysis to regulatory variants, since, for them, no change in translated protein is expected. Even for deep-intronic variants, it is important to evaluate their effect on splicing before studying their impact in gene expression. For intronic variants, a variety of in silico prediction tools are available, such as Splice Site Finder-like, MaxEntScan, NNSplice, GeneSplicer, and Human Splicing Finder.

Some new tools are beginning to address non-coding sequences [72,73,74]. As an alternative for non-coding variants, bioinformatics in silico analysis are useful to prioritize the variants that are located in DNase I, FAIRE peaks of open chromatin and TF consensus binding sites. Additionally, Information theory analysis has been used to evaluate if the binding strength of several TFs are predicted to be altered by *BRCA1/2* variants [75]. Moreover, the potential effect of a single nucleotide variant on RNA secondary structure can be tested by the prediction software SNPfold [76] and confirmed by the detection of covalent adducts in mRNA by the SHAPE assay [77].

Because polymorphisms are generally (but not necessarily) neutral, a first step to interpret the clinical significance of the variant is to determine its frequency in the population. Data about large populations and cohorts of HBOC patients are available for this purpose. If the variant is present in more than 1% of the general population, it is thus, on its own, thereby less likely to be high impact on disease risk, although it could still be a contributing factor. 

Co-occurrence with a pathogenic mutation is another important fact that can be extracted from BRCA databases. It is known that carrying a *BRCA* mutation in both chromosomes is embryonic lethal, therefore we can conclude that a variant identified in trans with a pathogenic variant in the same gene, without Fanconi anemia, is unlikely to be the causative mutation and is thus is classified as neutral. However, as non-coding variants reduce but do not abolish BRCA function, this approach should be applied with caution in this case.

The co-segregation of the variant in the affected individuals, in contrast to their absence in individuals without cancer is one of the stronger arguments for causality. However, performing co-segregation studies is challenging because the majority of VUS are often reported in a single family, which often leads to a lack of statistical power for the analysis, and non-coding variants are unlikely to be sufficiently penetrant to co-segregate with disease.

### 4.2. In Vitro Studies

Variants can potentially affect normal pre-mRNA splicing and be deleterious either via disruption of consensus sequences, creation of de novo sequences, or alteration of splicing regulatory elements [78]. Deep intronic variants can also impact splicing, such as through altering the function of branch sites, although the significance and mechanisms of such events remain unclear [79,80].

#### 4.2.1. Assays to Measure Splicing

The assays to evaluate the impact of VUS on RNA splicing focus on the gene region carrying the variant and compare the wild type with the variant sequencing providing proofs of the involvement of the variant in the splicing alteration. These assays complement the use of *in silico* prediction tools and they can be based either on a minigene construction or by an investigation of DNA transcripts derived from blood or tissue samples from patients performed by RT-PCR, qPCR, and droplet digital PCR [81]. During these experiments, the presence of both alleles can be considered as an indication of no effect of the VUS on splicing, whereas absence of the mutant allele in the full-length product can be an evidence of a complete effect. But, for RNA assays, quality control is an issue, as loss of splicing fidelity has been reported in cells analyzed under non-physiological conditions [82].

#### 4.2.2. Assays to Measure Interaction between Enhancers and Promoters

A series of chromosome conformation capture techniques have been developed to explore interactions between enhancers and promoters to start transcription. This includes: chromosome conformation capture (3C), circular chromosome conformation capture (4C), chromosome conformation capture carbon copy (5C), and high-resolution chromosome conformation capture (Hi-C) [83]. Chromosome conformation capture (3C) was used to evaluate the *c-Myc* gene where a variant in its distant enhancer has been shown to physically interact with the MYC locus, located 335kb away [35]. This approach has also been used to explore the impact of SNPs identified by genome-wide association studies (GWAS), related to the risk of ovarian and breast cancer [84]. For the moment no application has been described for the *BRCA1* and *BRCA2* genes, which opens new insights to explore unclassified non-coding variants.

#### 4.2.3. Assays to Measure Gene Expression and Protein Function (Functional Assays)

Functional assays can evaluate the variant’s impact on the ability of the protein to perform some key cellular functions, which in the case of non-coding variants, might be related to deficient gene expression. 

Luciferase reporter assay is a standard method to evaluate the impact of non-coding variants on gene expression. This assay consists of transfecting cells with a plasmid containing the luciferase gene under the control of DNA regulatory regions (promoter, enhancer, and repressor) with or without the variant of interest. The comparison between luciferase activities of cells transfected with the variant-containing plasmid and cells transfected with the plasmid containing the wild-type sequence, allow for the determination of the variant impact on the biological function of regulatory regions. This assay is also used to evaluate 3′UTR functional regions on gene expression. 

It is challenging to integrate calibrated functional assay data into multifactorial models, since pathogenic mutations do not affect the functional endpoints in the same way. Another issue is the low reproducibility between experiments, less prominent for variants with a greater effect. Plasmid DNA is placed in an artificial environment that may fail to reproduce the expression pattern of its endogenous equivalent due to differences on chromatin context. Regarding *BRCA1/2* non-coding variants, although the Luciferase assay is the current standard, the ideal cutoff that abrogates the allele expression has yet to be determined. For Lynch syndrome, it was suggested that 50% reduction of gene expression makes MMR function insufficient [85].

#### 4.2.4. Assays to Investigate the Underlying Mechanism of Variant Impact

Transcription factors (TF) and microRNAs operate via base-paring interactions with DNA and mRNA, respectively. The majority of TF binding sites are located in promoter, enhancer, and repressor elements (some of which overlap with the 5′UTR), while the majority of microRNAs binding sites are placed in 3′UTR. Some in silico tools are available to investigate whether the variant can create or disrupt one of these. For this purpose, microRNA and TF binding site prediction software, ENCODE ChIP-seq data, and information theory analysis can all provide clues that may be confirmed with in vitro experiments. 

In vitro experiments are generally the next step to elucidate the underlying mechanism through which the variant can interfere. For 3′UTR variants, the correspondent miRNA vector (synthetic or plasmid) is co-transfected with the Luciferase *BRCA1/2* 3′UTR reporter, with the variant or with the wild-type sequence. The results are then compared to determine if the variant has an impact. For promoter variants, several methods have been used for the characterization of protein-DNA interaction, including electrophoretic mobility shift assay (EMSA) [86] and Chromatin immunoprecipitation assays (ChIP) [87]. EMSA is based on the principle that a protein-DNA complex migrates more slowly through an electrophoresis gel than the corresponding free DNA. Differences in binding patterns between the wild type and mutant DNA sequences that labeled with a radioactive or luminescent tag, are indicative of TFs interacting with the DNA sequence in question. The candidate TF can be then identified by the use of an antibody against itself, using a ‘supershift’ assay. ChIP assays are an alternative method for directly visualizing an in vivo interaction between a specific protein and a regulatory element. After DNA cleavage by restriction enzymes, protein-DNA complexes are purified by immuno-precipitation with antibodies being directed against the protein of interest. Then, to confirm that the protein was linked to the TF binding site, the bound antibody is neutralized, proteins are digested, and DNA is analyzed for the presence of the regulatory element by PCR. Interacting proteins can also be identified using mass spectrometry.

Finally, promoter methylation has been described as an alternative mechanism of BRCA1 and BRCA2 silencing [56]. This is another mechanism of disrupting transcriptional regulation, which can be evaluated through pyrosequencing or Next Generation Sequencing.

### 4.3. Tumor Features

Tumors arising in *BRCA1* and *BRCA2* mutation carriers are different from each other and from tumors not associated with these mutations. More than 75% of breast cancers diagnosed in *BRCA1* mutation carriers are high grade and triple negative breast cancers. In contrast, the breast cancers of *BRCA2* pathogenic mutation carriers usually have a Luminal phenotype. A large study proved that histopathological features could predict the *BRCA* mutational status and led to the incorporation of pathological data into the algorithms for variants classification [88,89]. 

Loss of heterozygosis (LOH) analysis can also be useful. LOH is more frequently found in *BRCA*-mutation positive tumors than in sporadic tumors and it is generally related to the loss of the wild-type chromosome. The loss of the wild type *BRCA* allele usually corresponds to the ‘second hit’ and thereby adds an additional argument in favor of the variant pathogenicity.

Furthermore, other tumor characteristics such as a high genetic instability score, genome wide tumor methylation profile, evaluation of *PTEN* and *TP53* alterations, and gene expression arrays, could provide useful information. But, for the moment, except for tumor grade, hormonal receptor and HER2 status, this information is not incorporated into multifactorial likelihood models.

## 5. Impact of *BRCA1/2* Non-Coding Variants on Breast and Ovarian Cancer Predisposition

**Figure 1:**
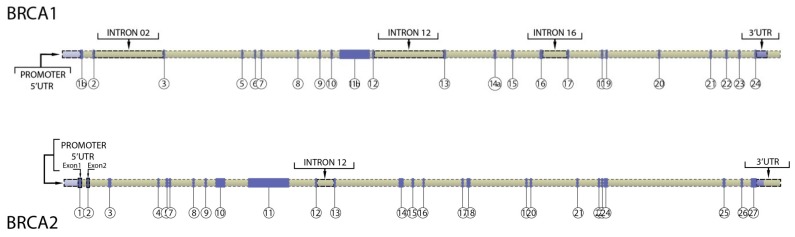
Non-coding regions of *BRCA1/2* genes studied to date.

Because *BRCA1/2* coding mutations only explain 10% of the predisposed families, exhaustive efforts have been undertaken for more than 20 years to identify other loci contributing to breast cancer susceptibility. It remains possible that some of the remaining risk may be related to the main HBOC genes *BRCA1/2*, potentially by variants causing the deregulation of expression. Until now, few studies have analyzed *BRCA1/2* non-coding regions (Figure 1 and Table 1).

Recent data originating from HBOC population screening confirm the presence of variants in *BRCA1/2* regulatory regions. Some of these variants are functionally active, which reinforces their possible link with hereditary predisposition (Table 2). But, for the moment, except for some non-coding variants that were identified in intron and exon boundaries with impact on splicing, all the sequence alterations identified in *BRCA1/2* non-coding regions remain unclassified. The incorporation of next generation sequencing analysis for germline tests should expand the availability of information, including a greater number of sequence variants whose biologic impact remains unknown.

We and others have screened *BRCA1* and *BRCA2* promoters of predisposed patients with no pathogenic variant identified, in search for potential 5′UTR mechanisms of gene deregulation [58,68,90]. The data generated from these studies led to the identification of some variants that demonstrated an impact on transcriptional regulation (Table 2). For some of these, the underlying mechanism of down regulation is related to disruption of interactions between transcription factors and their binding sites. While some variants are related to reduced promoter activity, others have been associated with increased gene expression. This latter effect is the opposite of what one would expect from a *BRCA1/2* variant associated with an increased breast/ovarian cancer risk. Nevertheless, these enhancing variants could inhibit some repressor elements localized within *BRCA1* and *BRCA2* promoters, thereby inducing an over expression of *BRCA1/2* [91]. We have seen that the *BRCA1/2* expression strongly fluctuates during the cell cycle. *BRCA1/2* expression is very low at the G1 phase to prevent DNA repair by homologous recombination at the wrong time. It can be hypothesized that variants leading to *BRCA1/2* overexpression could thus still perturb DNA repair mechanisms, thereby inducing genetic alterations causing cancer. Besides that, the inconsistent results that were occasionally observed when different cell-lines were used to evaluate the same variant may reflect the availability of transcription factors or co-factors among the cells and reinforce the utility of performing these tests in more than one cell line [92].

Promoter variants can also reduce gene expression through interference of CpG islands and consequent methylation-associated epigenetic silencing of the correspondent allele. Recently, this mechanism was described in two families carrying a *BRCA1* promoter variant (c.-107A>T). RNA sequencing revealed that the heterozygous variant that was segregated with the hypermethylated *BRCA1* allele, resulting in the allelic loss of *BRCA1* expression [83]. Similar to Lynch syndrome [90,91], this example raises the question of whether constitutional BRCA1/2 epimutations can represent an alternative mechanism for cancer predisposition. Considering that luciferase activity assay is ultimately indicative of both transcriptional and translational efficiency, it is noteworthy that, in functional studies, the reduced levels of BRCA1 protein is not always associated with reduced transcript levels [94,95]. Therefore, the disruption of post-transcriptional regulation should contribute in some cases. First, using RNAfold secondary structure prediction software, we could demonstrate that a *BRCA1* 5′UTR variant (c.-130del) impacts RNA conformation and it probably affects the binding of trans-acting factors and therefore mRNA translation [58]. This predicted effect was also described for some 3′UTR variants and a 5′UTR polymorphisms of *BRCA1*, both with an impact in translational efficiency [60,94].

A 5′UTR variant may also impact translation efficiency by interfering in the consensus motif for the start of protein translation. Wang et al. described a variant located two bases downstream *BRCA1* start codon that reduced the protein expression in this way. In the presence of the 5′UTR variant (+118A>T, c.-2A>T), luciferase activity was significantly reduced as compared to the wild type, while transcription efficiency and mRNA stability were assured by equal mRNA levels. Immuno-histochemical staining of the tumor could confirm the reduced expression of *BRCA1* protein for the variant carriers. Signori et al. also described a variant at position—3 from the *BRCA1* start codon associated with a significant decrease in mRNA translation through the same mechanism [95].

Germline variants have been described in the 3′UTR region of the *BRCA1/2* genes, some of them with a proven impact on gene expression [59,96,97]. MicroRNA is small non-coding RNA that negatively regulates mRNA translation by recognizing complementary sites, most located in this region. They can induce mRNA degradation or inhibit their translation resulting in gene down regulation. 3′UTR variants can disrupt pre-existing or create new cis-regulatory elements or binding sites for trans acting RNA binding proteins or micro-RNAs. However, there still exists a paucity of data on *BRCA1/2*3′UTRregions. Brewster et al. performed a screening of *BRCA1* 3′UTR in a large population of breast cancer cases with no *BRCA1/2* mutation that put in evidence 15 novel *BRCA1*3′UTR variants. One of them (c.*1340_1342del) related to the creation of a new microRNA binding site: miR-103. Another 3′UTR screening of 716 index cases that tested negative for *BRCA1/2* pathogenic mutations also detected SNPs and six rare variants in these regions, three of which are novel [60].

Though intronic data is even scarcer, a few intronic variants have been described by us and others. Two variants located in regulatory regions in the intron 2 and intron 12 sequences of *BRCA1* (c.81-3980A>G and c.4186-2022C>T, respectively) were able to revert the enhancing impact of these regions over *BRCA1* promoter activity. Although these regions are situated several kilobases downstream of the promoter region, it is hypothesized that they regulate *BRCA1* expression at the transcriptional level, most likely via gene looping [57,58].

For the moment it is difficult to predict the risk attributed to the presence of these variants, given the scarcity of data and the fact that they could have impact in different steps of gene expression, but, contrary to coding mutation, they may not impact protein function. Non-coding variants are expected to have more subtle quantitative effects and may probably be associated with a lower but still important impact on cancer risk. This impact on the relative risk of cancer is likely to occur in collaboration with other low, moderate, or high risk variants.

## 6. Clinical Practice Recommendations for Non-Coding Variants’ Carriers

There is currently no formal recommendation for classifying *BRCA1/2* non-coding variant carriers, nor guidelines for managing patients carrying these variants. As stated before, except for some variants that are located in the intron/exon transition with impact on splicing, the significance of nearly all variants that were identified in *BRCA1/2* non-coding regions remains uncertain. These sequence changes do not clearly affect the protein but cause subtle changes that are difficult to interpret. As a quantitative effect is expected, it is a major challenge to define a threshold that classifies the variant as causal or to determine their significance and contribution in breast/ovarian cancer susceptibility. So, it is still difficult to reach accurate conclusions that are useful for genetic counseling.

The last American College of Medical Genetics guideline provide no specific recommendation for the reporting and classification of variants that were identified in *BRCA1/2* promoters and intronic and untranslated regions [98]. To date, carriers should be managed exclusively based on their personal and family history which allows for the estimation of cancer risk. BOADICEA [99], BRCAPRO [100] and Tyrer-Cuzick [101] are examples of software-based models that are useful for estimating the risk of a woman developing cancer in the course of her life, regardless of *BRCA* status. Concerning breast cancer prevention, a life time risk >20% justifies intensive surveillance, including annual MRI and discussion of prophylactic surgery. However, available data is inadequate to support the use of chemoprophylaxis with tamoxifen and risk reducing salpingo-oophorectomy in this scenario. Those variants should therefore be included in a specific program for cosegregation and linkage analysis. Once these variants are unlikely to be sufficiently penetrant to co-segregate with disease, case control studies are very useful for assessing their impact.

Variants of uncertain significance constitute a challenge for the carriers and their doctors. They occur at a frequency between 5% for Caucasian Americans and up to 20% for African-Americans. In Europe, they are present in about 10% of *BRCA* screenings. Since the disease risk that is associated with the VUS is unknown, the risk is not interpretable, but it may be overinterpreted or misinterpreted. As a result, it should not be used for clinical decision. Little data is currently available about sequence changes in *BRCA1/2* non-coding regions. Even less information is available about the outcome of carriers that should be managed based on their lifetime cancer risk once their genetic screening remains inconclusive.

## 7. Conclusions

*BRCA1* and *BRCA2* remain the main candidates for explaining the high risk of cancer in HBOC syndrome. The first description of an epigenetic impact of a non-coding variant in *BRCA1* gene launches the necessity to continue the screening of *BRCA1/2* non-coding regions, in parallel with studies to determine their biochemical and clinical significance.

## Figures and Tables

**Table 1:** Priority regions of *BRCA1/2* genes for screening.

Region of Interest	Hg19 Coordinates	Length	Comments
*BRCA1* promoter	chr17: 41,277,500–41,278,500	1000 bases	Comprises 1 kb upstream on transcription start site
*BRCA1* 5′UTR (exon 1A)	chr17: 41,277,287–41,277,500	223 bases	Exon 1A
*BRCA1* 5′UTR (exon 1B)	chr17: 41,277,340–41,277,197	145 bases	Exon 1B
*BRCA1* 5′UTR + ATG (exon 2 to ATG)	chr17: 41,276,110–41,276,133	22 bases	5′ end of Exon 2
*BRCA1* intron 2	chr17: 41,271,250–41,272,100	850 bases	Includes validated enhancer and repressor elements that participate in gene looping and are conserved. Also contains sequences that UCSC/ENCODE indicates this region contains transcription factor binding sites, DnaseHS sites
*BRCA1* intron 12 (region 1)	chr17: 41,237,500–41,237,850	350 bases	UCSC/ENCODE indicates this region contains transcription factor binding sites, DnaseHS sites and is conserved.
*BRCA1* intron 12 (region 2)	chr17: 41,236,600–41,236,960	360 bases	UCSC/ENCODE indicates this region contains transcription factor binding sites, DnaseHS sites and is conserved.
*BRCA1* intron 16	Chr17: 41,220,900–41,221,250	350 bases	UCSC/ENCODE indicates this region contains transcription factor binding sites, DnaseHS sites
*BRCA1* 3′UTR (exon 24)	chr17: 41,196,311–41,197,698	1387 bases	From and including stop codon
*BRCA2* promoter	chr13: 32,888,616–32,889,616	1000 bases	Comprises 1 kb upstream on transcription start site
*BRCA2* 5′UTR (exon 1)	chr13: 32,889,616–32,889,805	189 bases	Exon 1 (Refseq)
*BRCA2* 5′UTR (exon 2 to ATG)	chr13: 32,890,558–32,890,600	42 bases	Includes translation start codon
*BRCA2* 3′UTR	chr13: 32,972,904–32,973,809	905 bases	From and including stop codon

**Table 2:** Population screening of *BRCA1/2* non-coding regions in patients tested negative for *BRCA1/2* codingmutations. A/Number of samples screened and number of variants identified. B/Variant impact on functional assays.

**A**					
**Gene**	**Region Screened**		**Population Screened (n)**	**Number of Samples Presenting a Variant**	**References**
*BRCA2*	Promoter		95	3	[68]
*BRCA1*	Promoter (255 bp)		3926	55	[58]
*BRCA2*	Promoter (380 bp)		3910	21	[58]
*BRCA1*	Intron 2 (326 bp)		3624	30	[58]
*BRCA1*	Intron 12 (360 bp)		2973	11	[58]
*BRCA1*	5′UTR		49	2	[93]
*BRCA1*	5′UTR		117*	2 (somatic)	[94]
*BRCA1*	5′UTR		96	1(somatic)	[95]
*BRCA1*	5′UTR (2400 bp)		6475	81	[90]
*BRCA2*	5′UTR (2000 bp		6603	60	[90]
*BRCA1*	3′UTR (1561 bp)		1612	7	[61]
*BRCA1*	3′UTR (1376 bp)		70	2	[62]
*BRCA1*	3′UTR (1382 bp)		716	5	[60]
*BRCA2*	3′UTR (902 bp)		716	1	[60]
**B**					
**Gene**	**Variant**	**Localization**	**Functional Test**	**Effect**	**References**
***BRCA1***					
*BRCA1*	c.-395C>T	Promoter	Luciferase assay	NS	[90]
*BRCA1*	c.-380G>A	Promoter	Luciferase assay	NS	[58,90]
*BRCA1*	c.-378C>A	Promoter	Luciferase assay	NS	[90]
*BRCA1*	c.-362T>G	Promoter	Luciferase assay	up regulation	[58]
*BRCA1*	c.-359G>T	Promoter	Luciferase assay	NS	[58]
*BRCA1*	c.-315del	Promoter	Luciferase assay	down regulation	[90]
*BRCA1*	c.-192T>C	Promoter	Luciferase assay	down regulation	[90]
*BRCA1*	c.-220C>A	Promoter	Luciferase assay	NS	[90]
*BRCA1*	c.-264T>G	Promoter	Luciferase assay	NS	[90]
*BRCA1*	c.-273G>A	Promoter	Luciferase assay	NS	[90]
*BRCA1*	c.-287C>T	Promoter	Luciferase assay	up regulation	[90]
*BRCA1*	c.-177C>T	Promoter	Luciferase assay	NS	[58]
*BRCA1*	c.-130del	Promoter	Luciferase assay	down regulation	[58]
*BRCA1*	c.-125C>T	Promoter	Luciferase assay	down regulation	[58]
*BRCA1*	c.-121G>C	Promoter	Luciferase assay	up regulation	[58]
*BRCA1*	c.-107A>T	Exon 1	Promoter methylation assays; RNA analysis by RT-PCR	down regulation	[93]
*BRCA1*	c.-71G>A	Exon 1	Luciferase assay	NS	[58]
*BRCA1*	c.-24T>C	Exon 1	Luciferase assay	NS	[58]
*BRCA1*	c.-3G>C (+117G>C)	Exon2	Luciferase assay; RNA translation assay	down regulation	[95]
*BRCA1*	c.-2A>T (+118A>T)	Exon2	Luciferase assay; RNA analysis by RT-PCR. Protein analysis by IHC	down regulation	[94]
*BRCA1*	c.81-3985A>T	Intron 2	Luciferase assay	up-regulation	[58]
*BRCA1*	c.81-3980A>G	Intron 2	Luciferase assay	down regulation	[58]
*BRCA1*	c.4186-2022C>T	Intron 12	Luciferase assay	down regulation	[58]
*BRCA1*	c.*291C>T	3′UTR	Luciferase assay	up-regulation	[61]
*BRCA1*	c.*528G>C	3′UTR	Luciferase assay	down regulation (MDAMB231) up-regulation (MCF7)	[61]
*BRCA1*	c.*713C>T	3′UTR	Luciferase assay	up-regulation	[60]
*BRCA1*	c.*718A>G	3′UTR	Luciferase assay	down regulation	[61]
*BRCA1*	c.*750A>G	3′UTR	Luciferase assay	NS up-regulation	[60,62]
*BRCA1*	c.*780C>T	3′UTR	Luciferase assay	up-regulation	[62]
*BRCA1*	c.*800T>C	3′UTR	Luciferase assay	NS	[61]
*BRCA1*	c.*1012A>G	3′UTR	Luciferase assay	NS	[62]
*BRCA1*	c.*1139G>T	3′UTR	Luciferase assay	up-regulation (MDAMB231) down regulation (MCF7)	[61]
*BRCA1*	c.*1271T>C	3′UTR	Luciferase assay	down regulation	[61]
*BRCA1*	c.*1286C>A	3′UTR	Luciferase assay	down regulation	[62]
*BRCA1*	c.*1340_1342del	3′UTR	Luciferase assay	up-regulation	[61]
***BRCA2***					
*BRCA2*	c.-492C>T	Promoter	Luciferase assay	NS	[68]
*BRCA2*	c.-467T>G	Promoter	Luciferase assay	up-regulation	[68]
*BRCA2*	c.-407G>A	Promoter	Luciferase assay	NS	[90]
*BRCA2*	c.-408T>A	Promoter	Luciferase assay	NS	[90]
*BRCA2*	c.-296C>T	Promoter	Luciferase assay	down regulation	[58,90]
*BRCA2*	c.-280_272dup	Promoter	Luciferase assay	up-regulation	[58]
*BRCA2*	c.-280del	Promoter	Luciferase assay	NS	[90]
*BRCA2*	c.-273G>T	Promoter	Luciferase assay	NS	[58]
*BRCA2*	c.-262G>A	Promoter	Luciferase assay	up-regulation	[68]
*BRCA2*	c.-248G>A	Promoter	Luciferase assay	NS	[68]
*BRCA2*	c.-220G>T	Exon 1	Luciferase assay	NS	[58]
*BRCA2*	c.-218G>A	Exon 1	Luciferase assay	NS	[58]
*BRCA2*	c.-213G>T	Exon 1	Luciferase assay	NS	[58]
*BRCA2*	c.-200C>T	Exon 1	Luciferase assay	NS	[90]
*BRCA2*	c.-197A>C	Exon 1	Luciferase assay	down regulation	[90]
*BRCA2*	c.-188C>T (+46C>T)	Exon 1	Luciferaseassay	NS	[68]
*BRCA2*	c.-175C>T	Exon 1	Luciferase assay	NS	[90]
*BRCA2*	c.-175C>T (+59C>T)	Exon 1	Luciferase assay	down regulation	[68]
*BRCA2*	c.-174G>A	Exon 1	Luciferase assay	up-regulation	[68]
*BRCA2*	c.-162G>A (+72G>A)	Exon 1	Luciferase assay	up-regulation	[68]
*BRCA2*	c.-159T>A	Exon 1	Luciferase assay	up-regulation	[68]
*BRCA2*	c.-133T>G	Exon 1	Luciferase assay	NS	[90]
*BRCA2*	c.-123G>A	Exon 1	Luciferase assay	up-regulation	[58]
*BRCA2*	c.-120G>A	Exon 1	Luciferase assay	up-regulation	[68]
*BRCA2*	c.-119A>G	Exon 1	Luciferase assay	down regulation	[68]
*BRCA2*	c.-94T>C	Exon 1	Luciferase assay	up-regulation	[68]
*BRCA2*	c.-87T>G	Exon 1	Luciferase assay	NS	[90]
*BRCA2*	c.-82G>C	Exon 1	Luciferase assay	NS	[90]
*BRCA2*	c.-77C>T	Exon 1	Luciferase assay	down regulation	[68]
*BRCA2*	c.-63C>T	Exon 1	Luciferase assay	NS	[68]
*BRCA2*	c.-52A>G	Exon 1	Luciferase assay	NS	[58]
*BRCA2*	c.-52A>G (+182A>G)	Exon 1	Luciferase assay	NS	[68]
*BRCA2*	c.*172 G>A	3′UTR	Luciferase assay	up-regulation (HBL-100) down regulation (MCF7)	[60]

* Information about BRCA1 coding sequencing not reported.
